# Conservation of *cis*-Regulatory Syntax Underlying Deuterostome Gastrulation

**DOI:** 10.3390/cells13131121

**Published:** 2024-06-28

**Authors:** Lorena Buono, Giovanni Annona, Marta Silvia Magri, Santiago Negueruela, Rosa Maria Sepe, Filomena Caccavale, Ignacio Maeso, Maria Ina Arnone, Salvatore D’Aniello

**Affiliations:** 1 Department of Biology and Evolution of Marine Organisms (BEOM), Stazione Zoologica Anton Dohrn, Villa Comunale, 80121 Naples, Italy; giovanni.annona@szn.it (G.A.); rosamaria.sepe@szn.it (R.M.S.); filomena.caccavale@szn.it (F.C.); ina.arnone@szn.it (M.I.A.); 2 Department of Research Infrastructure for Marine Biological Resources (RIMAR), Stazione Zoologica Anton Dohrn, Villa Comunale, 80121 Naples, Italy; 3 Centro Andaluz de Biología del Desarollo (CABD), Universidad Pablo de Olavide, 41013 Sevilla, Spain; mrtslvmgr@gmail.com; 4 Telethon Institute of Genetics and Medicine (TIGEM), 80078 Pozzuoli, Italy; s.negueruela@tigem.it; 5 Department of Ecosustainable Marine Biotechnology, Stazione Zoologica Anton Dohrn, Via Ammiraglio Ferdinando Acton, 80133 Naples, Italy; 6 Department of Genetics, Microbiology and Statistics, Faculty of Biology, University of Barcelona (UB), 08028 Barcelona, Spain; imaeso@ub.edu; 7 Institut de Recerca de la Biodiversitat (IRBio), University of Barcelona (UB), 08028 Barcelona, Spain

**Keywords:** *cis*-regulatory element, gene regulation, transcription factor, binding motif, Evo-Devo, ATAC-seq

## Abstract

Throughout embryonic development, the shaping of the functional and morphological characteristics of embryos is orchestrated by an intricate interaction between transcription factors and *cis*-regulatory elements. In this study, we conducted a comprehensive analysis of deuterostome *cis*-regulatory landscapes during gastrulation, focusing on four paradigmatic species: the echinoderm *Strongylocentrotus purpuratus*, the cephalochordate *Branchiostoma lanceolatum*, the urochordate *Ciona intestinalis*, and the vertebrate *Danio rerio*. Our approach involved comparative computational analysis of ATAC-seq datasets to explore the genome-wide blueprint of conserved transcription factor binding motifs underlying gastrulation. We identified a core set of conserved DNA binding motifs associated with 62 known transcription factors, indicating the remarkable conservation of the gastrulation regulatory landscape across deuterostomes. Our findings offer valuable insights into the evolutionary molecular dynamics of embryonic development, shedding light on conserved regulatory subprograms and providing a comprehensive perspective on the conservation and divergence of gene regulation underlying the gastrulation process.

## 1. Introduction

The functional and morphological characteristics of the cell are governed by complex interplays between the genome and molecular regulators orchestrating the expression levels of mRNAs and proteins. These intricate webs of interactions among diverse cellular components, called gene regulatory networks (GRNs), are essential for the regulation of cellular processes, tissue development, growth, and differentiation [1]. Since changes in these networks can result in significant shifts in the phenotype of an organism, affecting its morphology and functional development, they also play a key role in steering evolutionary trajectories.

The interactions between transcription factors (TFs) and *cis*-regulatory elements (CREs) form the backbone of these regulatory networks. TFs are proteins that recognize and bind to a specific collection of similar degenerated short genomic sequences known as transcription factor binding motifs (TFBMs). These TFBMs are often located in CREs, which represent functional regions of non-coding DNA that control the transcription of neighboring genes. CREs can be located within the immediate proximity of a gene or in more distal genomic regions. Usually, a single TF may bind to many CREs and, hence, control the expression of many genes. Concurrently, CREs can be bound by different TFs.

This configuration allows for a high degree of flexibility and complexity in gene regulation to ensure that the right transcripts are expressed in the right cells at the right time and in the right amounts throughout the life of the organism. When these motifs undergo alterations through mutational events, they can result in changes in gene expression, which, in turn, can lead to changes in the organism’s phenotype [2,3]. Hence, in the field of evolutionary developmental biology (Evo-Devo), TFBM syntax is crucial for understanding how genetic changes can drive developmental changes, as well as how these changes have evolved over time. For instance, the prevalence of certain motifs within different species suggests an evolutionary journey favoring similar developmental patterns. Concurrently, when these motifs appear in different genomic areas within the same species, they could indicate positive selection. This selection process involves specific DNA sequences recognized by TFs undergoing advantageous changes that enhance their binding affinity [1,4]. As a result, favorable motifs are more likely to persist or accumulate, influencing the regulation of gene expression and, in turn, affecting developmental patterns, potentially conferring adaptive advantages to the organism over time. Therefore, it is not surprising that those TFBMs serving as hubs for crucial developmental processes are often conserved across different species, reflecting the evolutionary importance of the gene-regulatory networks of which they are part.

GRNs exhibit an inherent hierarchical organization: some GRN subcircuits possess deep ancestral roots, while other subprograms are more recent and highly flexible. In a given species, evolutionary GRN adaptations are characterized by a combination of both the preservation and the alteration of regulatory dynamics and/or elements [5]. For example, studies on the evolution of GRN wiring in echinoderms have shown that, while there are differences in the organization of gene networks between sea stars and sea urchins, they also show subcircuit conservation and plasticity to some extent. This mosaic perspective on the evolution of GRN architecture suggests that while the overall structure of GRNs may vary between species, certain core programs are conserved, likely because subcircuits play a crucial role in key developmental processes [6].

Among the abovementioned key developmental processes, gastrulation certainly stands out as one of the most crucial and conserved embryonic stages in metazoans. As the developmental biologist Lewis Wolpert wrote in his book “*The Triumph of the Embryo*”: “It is not birth, marriage or death, but gastrulation, which is truly the most important time in your life” [7]. Indeed, during gastrulation, the fundamental embryonic architecture is established and the three germ layers (endoderm, mesoderm, and ectoderm) are specified, thereby forming the primitive body plan from which all animal forms arise, despite their evolutionary distance. Such pivotal developmental processes are usually underlined by modularized and complex GRNs, as these tend to exhibit greater stability over time than features controlled by simpler mechanisms [8]. Nevertheless, in line with the mosaic perspective on GRN evolution, there is also room for species-specific variation in the behavior and connections of these genes within their respective networks, reflecting the intrinsic dynamic essence of biological evolution [9]. Traditional investigation approaches have evolved based on the homology of expression patterns of individual genes between different species at the morphological level. Recent approaches rather emphasize the existence of complex network interactions that shape morphological and functional traits during development [8]. Although conserved developmental processes can differ in their fine regulatory mechanisms, the conservation of a core of TFs may be a testament to the fundamental roles that these proteins play during development. Therefore, despite the diversified evolution of complex genomic regulatory networks among species, it is possible to identify key TFs with largely conserved core functions. This conservation is crucial for the preservation of developmental processes across different species and phyla [10].

The present study aims to delve deeper into the regulatory genomic syntax of gastrulation as a universal step in the development of all metazoans. Taking advantage of comparative computational analysis of ATAC-seq datasets, which provide precise information on regulatory open chromatin, we explored the genome-wide blueprint of conserved TFBMs underlying gastrulation across four evolutionary paradigmatic species of deuterostomes: the echinoderm *Strongylocentrotus purpuratus*, the cephalochordate *Branchiostoma lanceolatum*, the urochordate *Ciona intestinalis*, and the vertebrate *Danio rerio*. We uncovered similarities and dissimilarities in TFBM enrichment, identifying a core of conserved TFBMs that may serve as keystones of gastrulation’s genetic programs. This comprehensive study provides a valuable perspective on how deuterostomes evolved and how their very early stages of development are coordinated, highlighting unexpectedly conserved regulatory subprograms and offering unprecedented broad evolutionary insight into the intricate landscape of gene regulation’s conservation and divergence.

## 2. Materials and Methods

### 2.1. Raw ATAC-Seq Data Retrieval 

Raw ATAC-seq data for the gastrula stages of the amphioxus *B. lanceolatum* (8 h post-fertilization at 18 °C) and the zebrafish *D. rerio* (6 h post-fertilization at 28 °C) were obtained from Marlétaz et al. [11]. The data for the urochordate *C. intestinalis* (6 h post-fertilization at 18 °C) and the sea urchin *S. purpuratus* (48 h post-fertilization at 15 °C) were downloaded from Madgwick et al. [12] and from Skvortsova et al. [13], respectively. These timepoints represent the gastrula stage in each species, a critical phase in embryogenesis characterized by extensive cell migration, the formation of the three primary germ layers (ectoderm, mesoderm, and endoderm), and the establishment of the body plan. Despite the variation in timing, these stages are homologous, providing a meaningful comparison for studying conserved and divergent mechanisms in early development across these diverse taxa. All of the data were obtained in biological duplicates. From here on, we will refer to each species simply as sea urchin, amphioxus, sea squirt, and zebrafish.

### 2.2. ATAC-Seq Analysis

FASTQ files were aligned against the respective reference genome (Table 1) using the bowtie2 v2.2.3 software [14] with the parameters *--no-unal --no-mixed --X 2000 --very-sensitive-local*. Although the sea squirt datasets came from *C. intestinalis* animals, the poor quality of the *C. intestinalis* genome assembly [15] led us to align these sequences to the closely related *Ciona robusta* genome, obtaining a good-quality read alignment, as previously described [12]. PCR artifacts and duplicates were removed using the rmdup tool, which is part of the SAMtools toolkit v1.7.1 [16]. The read start sites were offset by +4 and by −5 bp in the plus and minus strands, respectively, to determine the position where the transposase cuts the DNA. Then, read pairs with an insert <130 bp were selected, since they correspond to nucleosome-free reads [17]. Peaks were called using MACS2 [18] with the parameters *--nomodel --shift −45 --extsize 100* and the genome size of the corresponding organism. NarrowPeak output files from the MACS2 v2.1.2 step were used as inputs for the irreproducible discovery rate (IDR) [19]. Only peaks passing the IDR cut-off of 0.05 were considered for downstream analyses. Peaks were annotated using the script annotatePeaks.pl from the HOMER v4.11 toolkit [20], with default parameters. De novo motif enrichment and annotation of TFBMs in the sets of IDR peaks were performed using the script findMotifsGenome.pl from the HOMER v4.11 toolkit with the options *-p 4 -size given*. The complete HOMER outputs can be found in Supplementary Datasets S1–S4. DNA motifs that failed HOMER annotation for known TFBMs were reannotated with TOMTOM [21] using the database “vertebrates (in vivo and in silico)”. The matrix similarity among the 108 core TFBMs was calculated using TOMTOM. All of the TFBMs (and relative associated TFs) with a similarity q-value < 2 × 10^−5^ were grouped together, as shown in Table 2. 

### 2.3. Human Reactome Pathway Enrichment Analysis

Human Reactome Pathway enrichment was calculated with gProfiler (https://biit.cs.ut.ee/gprofiler/gost (accessed on 22 June 2024)) [22] using an unordered query list composed of the 62 TFs associated with the 108 conserved core TFBMs during the annotation step.

## 3. Results

### 3.1. Identification of Accessible Putative Regulatory Regions (APREs)

The initial aim of the present study was to identify species-specific gastrulation sets of CREs by analyzing raw ATAC-seq data through a standardized pipeline. This approach was designed to be applicable across all four examined species, intending to homogenize the analysis tools and parameters (see the Section 2 for further details). The objective was to reduce technical–analytical bias, ensuring that the outputs were optimized for direct comparison.

**Figure 1:**
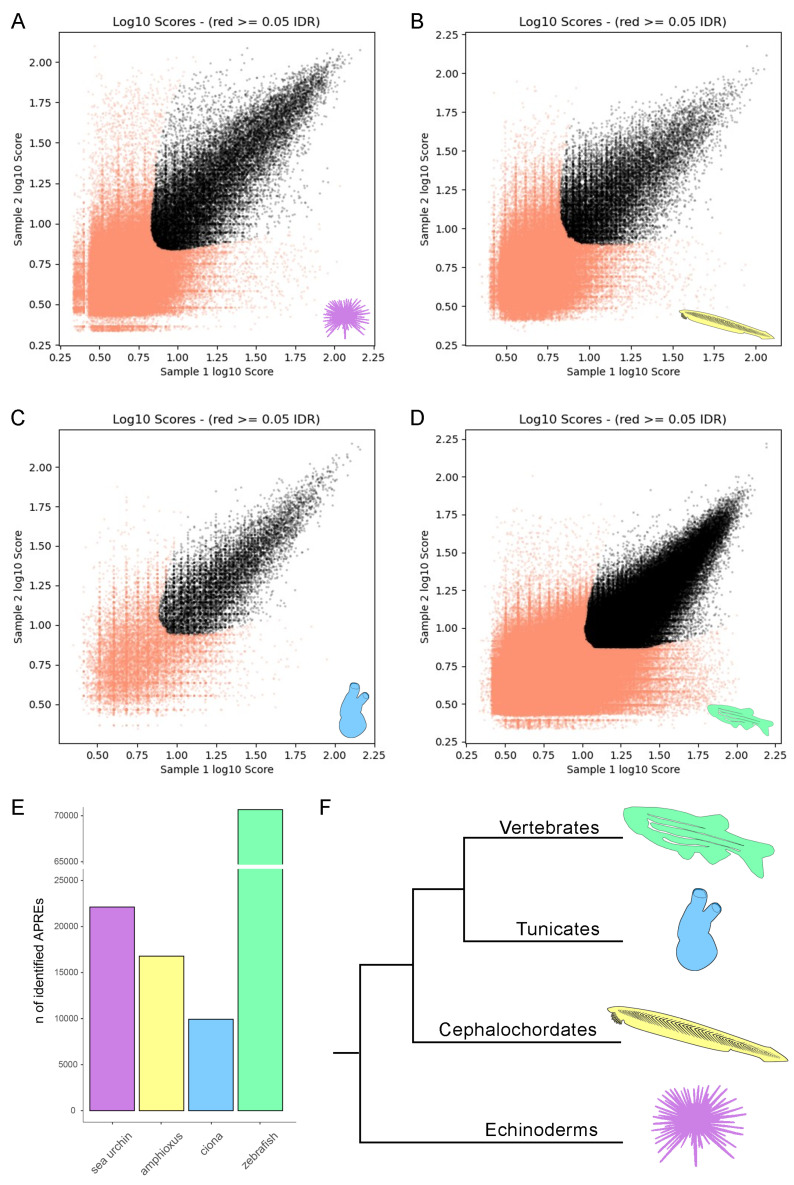
IDR plot of reproducible APREs in the sea urchin (**A**), amphioxus (**B**), sea squirt (**C**), and zebrafish (**D**). Bar plot indicating the number of highly reproducible APREs identified in each species (**E**). Phylogenetic tree showing the evolutionary relationships among the species examined in the present study. The tree was drawn based on deuterostome phylogenetic relationships as documented in Ref. [23] (**F**).

For each species, the ATAC-seq data were analyzed in biological duplicates. The IDR framework was used for each pair of peak callings to identify high-confidence peaks based on replicate information (Figure 1A–D). These peaks represent regions of open chromatin where the DNA structure is accessible, allowing molecular interactions such as TF binding. This information is crucial for characterizing the position and contents of genomic regulatory elements that modulate gene expression. Hereafter, we will refer to these sets of high-confidence peaks as accessible putative regulatory regions (APREs). In the gastrulae of the sea urchin, amphioxus, sea squirt, and zebrafish, we identified 22,119, 16,786, 9931, and 70,659 high-confidence peaks, respectively (Figure 1E). The high-confidence peaks represent 13.5%, 13.9%, 33.9%, and 18.2% of the total ATAC-seq peaks in each organism, respectively. Although the gastrula of the vertebrate zebrafish exhibited the greatest number of identified APREs, its closest chordate relative, the sea squirt, had the lowest count, differing by nearly an order of magnitude. The echinoderm sea urchin ranked second in terms of the number of APREs, followed by the cephalochordate amphioxus. Notably, the observed number of APREs across species during gastrulation not only varied remarkably but also did not align with their respective evolutionary relationships (Figure 1F).

### 3.2. Characterization of the APRE Distribution

**Figure 2:**
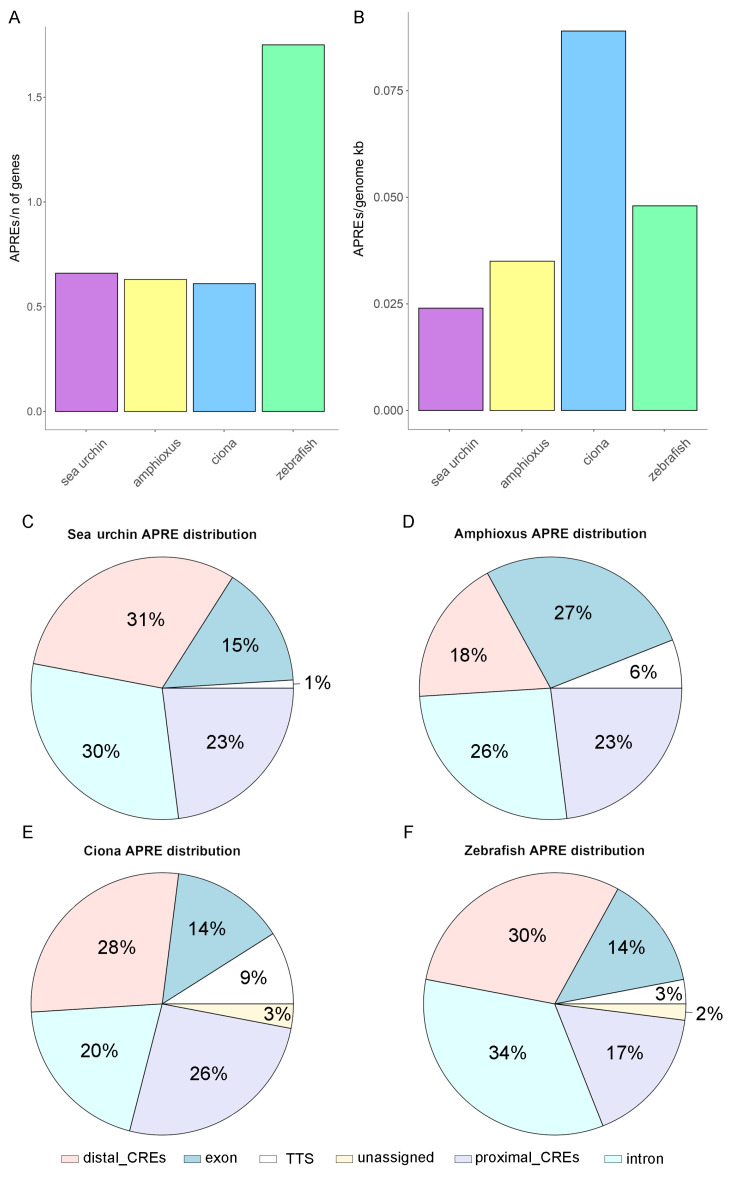
The ratio between the number of identified APREs and the number of genes annotated in each species (**A**). The ratio between the number of identified APREs and the genome size of each species (**B**). Genomic feature distribution of APREs in the sea urchin (**C**), amphioxus (**D**), sea squirt (**E**), and zebrafish (**F**).

To further investigate the correlations between gastrula APRE amount and evolutionary relationships, we normalized the APRE counts relative to the total number of genes and the genome size within each species. Remarkably, our findings indicate that the APRE per gene ratio of zebrafish was nearly three times greater than that of the other examined species (1.75, compared to 0.61 in the sea squirt, 0.63 in the amphioxus, and 0.66 in the sea urchin) (Figure 2A). These results, which are specifically focused on gastrulation, align with a recent comprehensive analysis by Marlétaz and colleagues [11], revealing a greater abundance of APREs per gene in vertebrates than in amphioxus. Surprisingly, our data further showed that the APREs per gene ratio remained relatively constant not only between the two analyzed non-vertebrate chordate species but also in sea urchins. This suggests that the increase in regulatory elements per gene represents a distinctive characteristic of vertebrates, potentially unrelated to their different body symmetries, the acquisition of the notochord, and more complex organ systems. The density analysis of APREs in relation to genome size failed to reveal a clear evolutionary correlation (see Section 2 for further details about genome sizes). Notably, the sea squirt, which is well known for having one of the most compact chordate genomes [24], exhibited the highest APRE density (0.089 APREs/genome Kb). In comparison, the remaining chordates (zebrafish and amphioxus) exhibited APRE densities of 0.048 and 0.035 APREs/genome Kb, respectively (Figure 2B). Strikingly, the sea urchin displayed the most dilated gastrula regulatory landscape, characterized by the lowest density, with 0.024 APREs per genome Kb (Figure 2B).

Finally, we associated APREs with the genomic features of their corresponding species genome annotation. This step involved identifying neighboring genes and determining whether the peaks coincided with specific genomic features, including proximal CREs (promoter-TSS; defined from −1 kb to +100 bp by default), transcription termination sites (TTS; defined from −100 bp to +1 kb by default), exons, introns, or distal intergenic CREs. Following APRE annotation, we computed the percentage of APREs within each type of genomic feature, enabling cross-species comparisons. Our findings revealed a relatively uniform APRE distribution across different genomic features for the four species, except for the amphioxus, which exhibited a significantly greater percentage of APREs located in exons (27%, compared to 15%, 14%, and 14% in the sea urchin, sea squirt, and zebrafish, respectively), at the expense of distal enhancers (18%, versus 31%, 28%, and 30% in the sea urchin, sea squirt, and zebrafish, respectively; *p*-value = 0.009, calculated with Dixon’s test) (Figure 2C). The overall increase in APREs within exons at the expense of distal intergenic CREs appears to be a distinctive trait specific to amphioxus, which is not shared by the phylogenetically close sea squirt or sea urchin. Indeed, the percentage of APREs in distal intergenic regions in the sea squirt and sea urchin mirrors the percentage registered in zebrafish (Figure 2C), even if they tend to have different distances to the TSS. Once again, the sea squirt showed the most compact genome and APRE distribution, with a median distance of intergenic APREs from the TSS of 3454 nucleotides, followed by the amphioxus (7378 nucleotides), sea urchin (12,132 nucleotides), and finally the zebrafish (23,198 nucleotides) (Supplementary Figure S1). Although a globally longer distance between APREs and TSSs has already been observed in vertebrates compared to amphioxus [11], our findings suggest that APRE density and distribution during gastrulation do not strictly follow expectations based on overall phylogenetic relationships and genome features, highlighting nuanced regulatory differences among species despite their evolutionary proximity. However, it is important to consider that some of these differences may be influenced by variations in the quality of gene annotations. For instance, in some species, the availability of extensive RNA-seq datasets might have contributed to a more exhaustive collection of annotated exons, particularly in regions such as UTRs and lowly expressed non-coding RNAs. Therefore, further investigations may be necessary to elucidate the underlying factors contributing to the observed CRE landscape variations across different taxa.

### 3.3. Transcription Factor Binding Motifs Analyses 

**Figure 3:**
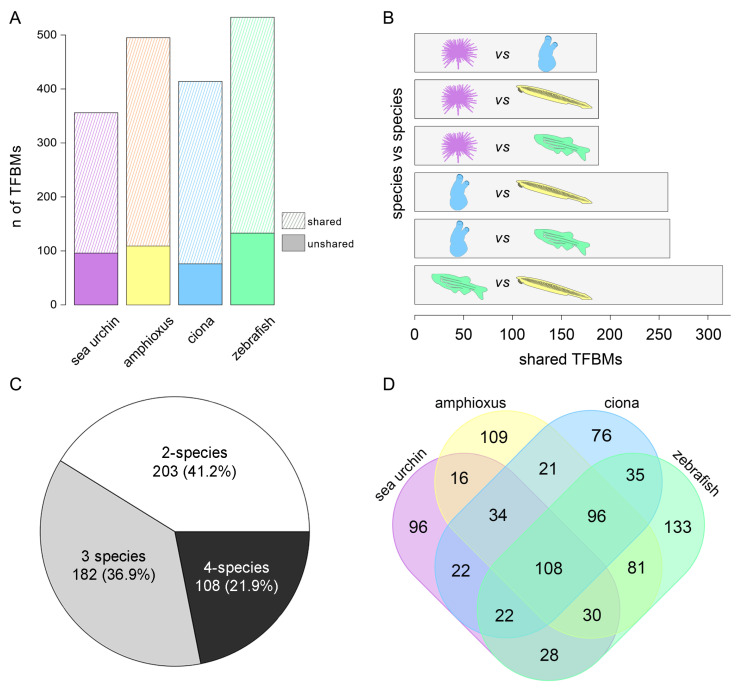
The number of de novo identified TFBMs in each species. The solid color indicates TFBMs identified only in that species, while the dashed color indicates TFBMs shared with at least one other species (**A**). Numbers of shared TFBMs in one-to-one comparisons (**B**). Percentages of shared TFBMs by 2, 3, or 4 species (**C**). Venn diagram indicating the detailed numbers of TFBMs shared among species (**D**).

To enhance our understanding of the *cis*-regulatory DNA syntax in the four deuterostome species investigated, we identified enriched DNA motifs within each of the four sets of APREs and associated them with known TFBMs through a multi-database approach (see the Section 2 for further details). We found 356, 495, 414, and 533 distinct enriched motifs in the sea urchin, amphioxus, sea squirt, and zebrafish, respectively (Figure 3A). Among the non-vertebrate chordates, the amphioxus exhibited the greatest number of enriched TFBMs, closely resembling the result in the zebrafish rather than that in the sea squirt. Subsequently, we analyzed the proportion of TFBMs shared with at least one of the other species under investigation. Our findings indicate that, in the sea urchin, amphioxus, sea squirt, and zebrafish, 73%, 78%, 81.6%, and 75% of the motifs are shared with other organisms, respectively (Figure 3A). Upon evaluating the general shared quota of TFBMs, we also conducted one-to-one comparisons between species. Our analysis revealed a lower number of common sea urchin TFBMs than those of the other three organisms. However, the incidence of shared TFBMs was notably consistent: 188 with the amphioxus, 186 with the sea squirt, and 188 with the zebrafish. The number of TFBMs shared by the sea squirt and amphioxus (259) was greater than that shared by the sea squirt and zebrafish (261). Ultimately, among all of the non-vertebrates analyzed, the amphioxus displayed the highest level of similarity with the zebrafish, with a shared count of 315 TFBMs (Figure 3B). 

In summary, our comprehensive findings revealed a total of 493 shared TFBMs. Among these, 203 (41%) were shared exclusively between two organisms, 182 (37%) were shared among three organisms, and 108 (22%) were shared among all four species examined (Figure 3C). Interestingly, the sharing of DNA motifs does not strictly adhere to phylogenetic relationships. For instance, we identified 28 shared motifs between the sea urchin and zebrafish without concurrent presence in the amphioxus and sea squirt. Additionally, in cases where the TFBMs were shared by three species, we occasionally found the excluded organism to be positioned in the middle of the evolutionary tree. For example, 22 motifs were shared among sea urchin–sea squirt–zebrafish, and 30 motifs were shared among sea urchin–amphioxus–zebrafish (Figure 3D). It remains to be demonstrated whether these results are due to resolution problems and/or variability in the techniques used, loss of specific motifs in particular organisms, or divergent evolutionary phenomena. Nevertheless, the presence of 108 motifs enriched in CREs during gastrulation across all four species is worth mentioning (the complete list of shared TFBMs can be found in Supplementary Dataset S5). This intriguing finding suggests the existence of a core regulatory syntax and potentially conserved TF functions throughout the evolution of deuterostomes, spanning from echinoderms to vertebrates.

### 3.4. Identification of a Conserved Core of Transcription Factor-Binding Motifs during Gastrulation

Our computational approach enabled the identification of a core of 108 putative DNA binding motifs shared among the selected representatives from each considered phylogenetic group. Due to motif syntax redundancy, these 108 motifs could be collapsed and successfully linked to 62 known transcription factors (Table 2). This redundancy arises because TFs within the same family often share highly conserved DNA-binding domains that recognize similar or overlapping nucleotide sequences [25,26]. Such overlapping of TFBMs’ syntax allows different TFs of the same family to bind the same genomic regions, forming a robust and versatile regulatory network. This redundancy supports a fine-tuned regulation through combinatorial and cooperative interactions among TFs, while also enhancing the precision and adaptability of cellular responses to various stimuli. Additionally, the overlapping of TFBMs can compensate for the loss or mutation of individual TFs, ensuring the fidelity of gene expression under diverse conditions [27,28]. To manage this syntax redundancy, motifs with high sequence similarity and their corresponding TFs were grouped together. We further validated the effective expression of the TFs associated with TFBMs during the gastrula stage in zebrafish, using RNA-seq data from Marlétaz et al. (2018) [11]. Our analysis revealed that 53 out of the 62 TFs linked to TFBMs in Table 2 are robustly expressed during gastrulation. For the remaining 9 TFs that were not expressed, we observed the concomitant expression of other TFs from the same family that likely share the same TFBMs (FoxA1, FoxL2, FoxF1, Sox10, Nr2E1), suggesting that the presence of functionally similar TFs can possibly compensate for the lack of expression of specific family members, ensuring the necessary regulatory functions during this critical developmental stage. The absence of expression of the remaining TFs (Myf5, MyoG, Dec2, and Gf1b) may be attributable to several factors, including the asynchronous chromatin remodeling, which could precede or follow the actual transcriptional expression of these TFs. Another possibility could be the incomplete or inaccurate annotation of gene models and TFBMs, which can lead to the misidentification of the TFs linked to precise TFBMs.

**Figure 4:**
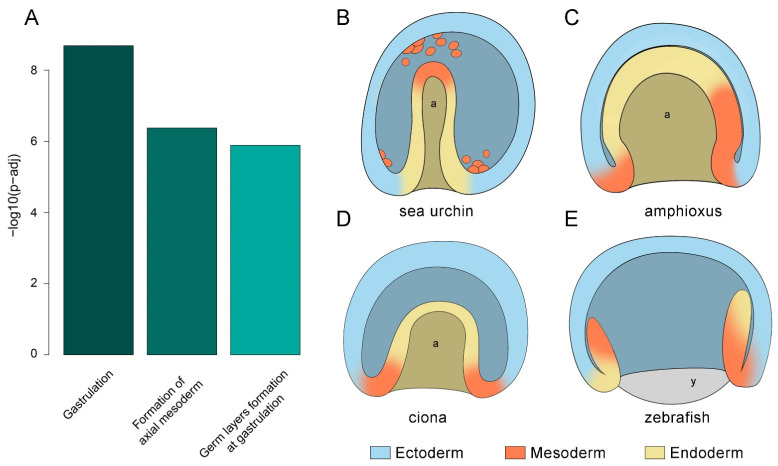
Enriched human Reactome pathway resulting from the 62 core TFs; the term ranked #2 (deactivation of the beta-catenin transactivating complex) was omitted; the complete list of terms can be found in Supplementary Dataset S6 (**A**). Stylized outline of the gastrula of the sea urchin (**B**), amphioxus (**C**), sea squirt (**D**), and zebrafish (**E**). a = archenteron; y = yolk.

Strikingly, the analysis for human Reactome pathway enrichment of the 62 core TFs yielded the most enriched terms related to gastrulation and the formation of the three germ layers [29] (Figure 4A; the complete list of enriched human Reactome pathways and involved TFs can be found in Supplementary Dataset S6). These findings reveal surprising conservation in the regulatory landscape of the gastrulation process, despite the significant evolutionary distance since the split from the common deuterostome ancestor, the different morphologies of the considered gastrulae (Figure 4B–E), and the distinct adult body plans. As corroborated by the pathway enrichment analysis, such conservation could potentially extend to the human species.

Notably, our results highlight the significant involvement of TFs with well-established functions in gastrulation (Table 2), such as those from the Forkhead [30,31,32,33,34,35], Homeobox [35,36,37,38,39,40,41,42], Hmg [43], and bHLH families [44,45]. At a general level, the main roles of these key TFs in gastrulation include the organization of the future body plan, neuroectoderm specification, and progenitor state maintenance. During organogenesis, the same genes cooperate for the specification and development of a variety of organs, such as the gut, lungs, liver, kidneys, pancreas, gonads, heart, and eyes, in addition to participating in myogenesis, angiogenesis, vasculogenesis, spermatogenesis, and oogenesis (Table 2). Forkhead (Fox) is an ancient class of TF that is a very well-known regulator of gastrulation, stem cell maintenance, and cell-cycle control and is required for the normal specification, differentiation, maintenance, and function of several organs [31,32,33,34,35,39,46]. Similarly, Homeobox TFs, such as Otx genes [36], Gosecoid [40,41], and Irx [35,38,39], have widely reported functions during gastrulation. High-mobility group (Hmg) TFs, particularly the Sox family, are also among the earliest classes of genes expressed during embryonic development, and they regulate progenitor cell specification and the terminal differentiation of multiple cell types [47].

**Table 2:** This table indicates the main transcription factor families found in gastrulation APREs, with information related to embryonic layers and their roles in gastrulation and organogenesis. The associated TFs that are expressed in zebrafish RNA-seq gastrulae are displayed in black, while the missing TFs (RNA counts < 10) are displayed in grey. The circadian clock genes (bHLH and bZip families) are highlighted in pale yellow, the genes forming the AP-1 complex are highlighted in green, and the genes in the WNT signaling pathway are highlighted in orange.

Binding Motif Sequence	TF Family	TF	Germ Layer Pathway	Known Role in Gastrulation	Role in Organogenesis	Refs.
WAAGTAAACA	Forkhead	FoxA1	Endoderm Mesoderm	Specification of anterior ectodermal program and repressor of posterior fates	Specification and differentiation of endodermal structures: gut, lungs, liver, kidneys, pancreas, prostate, notochord, nodes	[30,31,34,35,39,46,48,49,50,51,52,53]
CYTGTTTACWYW		FoxA2	Ectoderm			
BSNTGTTTACWYWGN		FoxA3	Endoderm Mesoderm			
NVWTGTTTAC		FoxK1	Mesoderm		Myogenesis	
SCHTGTTTACAT		FoxK2				
WWTRTAAACAVG		FoxL2	Mesoderm Ectoderm		Specification of ovaries and eyes	
DGTAAACA		FoxO3	Mesoderm		Vasculogenesis (blood vessels)	
TRTTTACTTW		FoxM1	Ectoderm	Activation of G2-M cell-cycle regulators	Neuronal differentiation	
WWATRTAAACAN		FoxF1	Mesoderm		Lung, liver, and gut development	
KTGTTTGC		FoxJ2			Angiogenesis, spermatogenesis, ciliogenesis	
CTGTTTAC		FoxO1	Mesoderm		Vasculogenesis (blood vessels)	
NYYTGTTTACHN		FoxP1	Ectoderm		Specification of nervous system and heart	
TTRAGTGSYK	Homeobox	Nkx3-2	Endoderm Mesoderm	Neuroendoderm specification	Development of embryonic skeletal system and intestinal epithelium	[36,37,38,39,40,41,42,54,55,56,57,58,59,60,61,62,63,64,65,66,67,68]
VNNGGATTADNN		Gcs	Endoderm Mesoderm	Head organizer, notochord formation, cell migration	Anterior brain induction, craniofacial development, head patterning	
WACACGTAACTT		Irx3	Ectoderm Mesoderm	Organizer, specification of neuronal progenitor cells of the spinal cord, anteroposterior patterning of neural axis	SHH-dependent neural patterning, development of inner ear, limbs, heart, kidneys, and facial/gill cartilage (regulated by the Wnt pathway)	
NGTGTTCAVTSAAGCGKAAA		Pax6	Ectoderm Mesoderm	Specification of neuronal progenitor cells of the spinal cord	Development of eyes, pancreas, nose, nervous system, and pituitary gland	
RTGATTKATRGN		Pbx2	Mesoderm		Development of hindbrain, tectum, retina, axial skeleton, and thyme	
SCTGTCAVTCAV		Pknox1	Mesoderm	Apoptosis, chick primitive streak formation, (novel) regulator of EMT	Adipogenesis, differentiation of hematopoietic precursor cells, hindbrain segmentation, head cartilage development	
NYTAATCCYB		Otx2	Ectoderm Endoderm	Specification of neuroectoderm and endoderm	Development of eyes, nose, ears, nervous system, first pharyngeal arch formation, and midbrain–hindbrain border (MHB) induction (regulated by retinoic acid signaling)	
KACACGTCTCTY	bHLH	Hey2	Mesoderm		Cardiovascular development (Notch signaling)	[44,45,69,70,71]
VVCCACGTGG		c-Myc	Mesoderm Extraembryonic tissues	Cellular plasticity maintenance		
VRCCACGTGG		n-Myc				
BAACAGCTGT		Myf5	Mesoderm		Myogenesis	
AACAGCTG		MyoG				
DGCACACGTG		Mnt				
WNBCACGTGA		Arntl1		Chick primitive streak, mediates hypoxia-induced IGFBP-1 expression	Development of nervous system, optic vesicles, notochord, foregut, and somites	
GHCACGTG		Clock		Chick primitive streak	Development of nervous system, optic vesicles, notochord, foregut, somites, and heart	
KCCACGTGAC		Npas2				
KCACGTGMCN		Dec2	Prechordal plate		Neural crest cells	
VTTACGTAAYNNNNN	bZip	Nfil3			Immune system	[72,73]
RTTATGYAAB		Hlf			Clock-controlled gene, never associated with gastrulation	
VTGACTCATC		AP-1				
GATGASTCATCN RATGASTCAT		JunB			Innate and adaptive immune responses, tumorigenesis	
NNATGASTCATH		Fra1			Differentiation of adipocytes, chondrocytes, and osteoblasts; placental vascularization	
GGATGACTCATC NATGASTCABNN		FosL2			Cell proliferation and differentiation	
	
DATGASTCATHN		Atf3	Mesoderm			
DATGASTCAT		Batf			Differentiation of immune system cells	
NKGMCACGTGDCMNN		Creb3L2			Chondrogenesis, regulation of the secretory pathway	
RRTSACGTSD		Usf2				
BCCATTGTTC	HMG	Sox2	Ectoderm Endoderm	Development of nervous system, progenitor state maintenance	Embryonic stem cell pluripotency; specification of nervous system and eyes	[43,47,74,75,76,77,78,79,80,81,82,83]
CCWTTGTY		Sox3	Ectoderm Endoderm	Nervous system development, progenitor state maintenance	Formation of hypothalamic–pituitary axis, suppression of neuronal differentiation, craniofacial morphogenesis, sex determination	
YCTTTGTTCC		Sox4		Regulator of epithelial–mesenchymal transition (EMT)	Development of eyes, pancreas, and skeletal system; differentiation of noradrenergic neurons	
CCATTGTTNY		Sox6	Ectoderm Endoderm Mesoderm		Development of nervous system, chondrogenesis, maintenance of cardiac and skeletal muscle cells	
CCWTTGTYYB		Sox10		Specification of neural crest	Development of neural crest and peripheral nervous system; glia and melanocyte development	
RAACAATGGN		Sox15			Inhibition of myoblast differentiation, skeletal muscle regeneration, regulation of stem cell pluripotency, germline development	
AGGVNCCTTTGT		Sox9	Endoderm	Specification of neural crest	Chondrocyte, otic placode and glial differentiation, development of skeletal system, inner ear, craniofacial, male sex determination	
ASWTCAAAGG		Tcf3	Endoderm	Axis specification, regulator of pluripotency	Neuronal differentiation, mesenchymal–epithelial transition (MET), eye development	
ASATCAAAGGVA		Tcf4	Mesoderm		Neuronal differentiation	
CCTTTGATST		Lef1	Ectoderm			
CTTTGATGTGSB		Tcf7	Mesoderm		T-cell lymphocyte differentiation	
CTGTCTGG	MAD	Smad2	Endoderm	Specification of the anterior primitive streak, dorsoventral axis specification		[84,85,86,87]
VBSYGTCTGG		Smad4	Endoderm	Specification of the anterior primitive streak	Heart and skeletal muscle development	
BGTTGACTWH	NR	Nr2E1			Anterior brain differentiation, eye (retinal) development	[23]
CGTTGACTWW NCGTTGACTT		Nr2F1			Neurogenesis (regulated by retinoic acid signaling)	
NNNTTGACYWNNNNN		Nr4A1	Endoderm Mesoderm		Regeneration, immune response	
YACGTMAY	Zinc Finger	Atf1		Role in correct gastrulation	(regulated by Notch signaling)	[88,89,90]
CCTGCTGAGH		Zic	Ectoderm	Left–right axis formation	Early neurogenesis (BMP signaling)	
MAATCACTGC		Gfi1b	Mesoderm		Hematopoiesis	
GGCGGCTG		Znf460				
Several BMs		Yy1 Yy2	Embryonic and extraembryonic tissues	Morphogenetic movements	Cardiac morphogenesis (regulated by nodal signaling)	
HNACGCTCCT		Klf13			Heart development	

However, in some instances, the exact role of certain TFs in the gastrulation process remains unclear. This is notably the case for TFs associated with the circadian rhythm, such as Clock, Bmal, Npas, and Nfil, known as key components of the internal clock in all organisms, regulating biological cycles in response to changes in environmental light.

## 4. Discussion

Our thorough examination of deuterostome CREs during gastrulation revealed remarkable divergences and similarities in regulatory landscapes between the sea urchin, amphioxus, sea squirt, and zebrafish. Our results revealed that zebrafish exhibit a more similar regulatory profile to amphioxus, in comparison to the phylogenetically closer sea squirt. This may reflect the rapid evolution of the urochordate genome [91] and, on the contrary, the genomic stasis of cephalochordates that are more similar to the chordate ancestor’s genome and, probably, to modern vertebrates [92]. The nuanced parallels, particularly pronounced in the zebrafish–amphioxus comparison, encompass both the characteristics of APRE landscapes and TFBM complexity. These findings challenge conventional expectations in Evo-Devo, suggesting the presence of a regulatory syntax that is specifically conserved within vertebrates and cephalochordates. Furthermore, the discovery of a substantial number of shared DNA binding motifs among the four species suggests a conserved regulatory logic that is crucial for the organization and specification of the three germ layers across all deuterostomes. In our opinion, this is an extraordinary finding, considering that the representative species chosen in the present work arose from a common deuterostome ancestor at approximately 590–600 mya [93,94], and such temporal divergence is also reflected in distinct adult body plan outputs.

While some results were anticipated, such as the identification of binding motifs for transcription factors with a well-established role in gastrulation, others were less expected. We obtained signals from TFs with putative active roles in gastrulation, even though their involvement in this stage of development has not been previously reported and is more commonly documented during organogenesis. These findings imply that certain TFs may have earlier expression and functions during development than previously recognized, a finding made possible through the comparative analysis of active chromatin that we performed. However, it should be mentioned that chromatin opening may in some cases simply precede the actual expression and function of the controlled genes, as in the case of poised enhancers [95].

Another interesting aspect is that, despite some studies attempting to clarify the role of circadian genes during gastrulation, it remains unclear whether a functional regulatory clock already exists during gastrulation in deuterostomes, or whether the putative TFs initially perform other transcriptional actions and are subsequently recruited into a proper regulatory circadian clock. Several studies have suggested that a functional circadian clock matures predominantly after organogenesis is completed [96]. In fact, the circadian clock appears to be dispensable for normal embryogenesis in chickens, frogs, and zebrafish. However, it has been shown that many clock genes are expressed from the earliest stages of development in these species. For instance, during early chicken embryo development, Bmal1 and Clock are expressed in a ‘‘salt and pepper’’ pattern in the primitive streak and the Hensen’s node, indicating the existence of asynchronous oscillatory transcription with potential functions independent of light and darkness [70]. Similarly, Clock is expressed at very early *Xenopus* gastrula stages in the Spemann’s organizer and is localized to the mesodermal cells of the dorsal blastopore lip and the ectodermal cells above. As gastrulation progresses, Clock becomes confined to the anterior neuroectoderm [97].

Ultimately, this work reveals the necessity of further exploring the regulatory networks associated with the identified highly conserved TFs in different organisms. Although a precise blueprint for each TF would be useful to unravel their species- and stage-specific regulatory programs, so far, the current limitations are represented by the scarce availability of specific antibodies for target TFs in less conventional model organisms, hindering in-depth studies utilizing ChIP-seq experiments. However, with the continuous decrease in sequencing costs and the persistent evolution of technology, notable innovations such as DamID-seq have emerged as promising tools. DamID-seq, a NGS-based methodology, facilitates the identification of DNA–protein binding regions through the use of a methyltransferase fusion protein [98]. The evolution of cutting-edge techniques coupled with advances in bioinformatics presents an exciting opportunity to overcome previous experimental constraints and further explore the roles played by conserved TFs during the gastrulation processes of diverse species.

## 5. Conclusions

This study highlights the remarkable conservation of a core of TFBMs underlying gastrulation among four selected deuterostome organisms that split from a common ancestor approximately 600 mya. These results offer a novel evolutionary perspective on how the *cis*-regulatory landscape steers one key developmental process: gastrulation. In the future, it would be very challenging and informative to include ATAC-seq data from protostomes in such comparative analyses, in order to uncover the degree of conservation and to infer the ancestral regulatory state of bilaterian gastrulae.

## Figures and Tables

**Table 1:** Reference genomes considered in the present work.

Species	Genome Assembly	Genome Size
*Strongylocentrotus purpuratus*	GCF_000002235.5	9.21 × 10^8^
*Branchiostoma lanceolatum*	GCA_927797965.1	4.74 × 10^8^
*Ciona intestinalis*	GCA_000224145.1	1.12 × 10^8^
*Danio rerio*	GCF_000002035.5	1.46 × 10^9^

## Data Availability

The original contributions presented in the study are included in the article/Supplementary Material, further inquiries can be directed to the corresponding authors.

## Supplementary Materials

The following supporting information can be downloaded at https://www.mdpi.com/article/10.3390/cells13131121/s1, The raw ATAC-seq data used in this study are available in publications cited in the Section 2. The resulting files are available as Supplementary Datasets.
