# Supplementary figures and images for: Conservation of cis-Regulatory Syntax Underlying Deuterostome Gastrulation
Source: Cells. 2024 Jun 28;13(13):1121. doi: 10.3390/cells13131121 (PMC11240583; doi:10.3390/cells13131121)

Organism sea urchin amphioxus sea squirt zebrafish

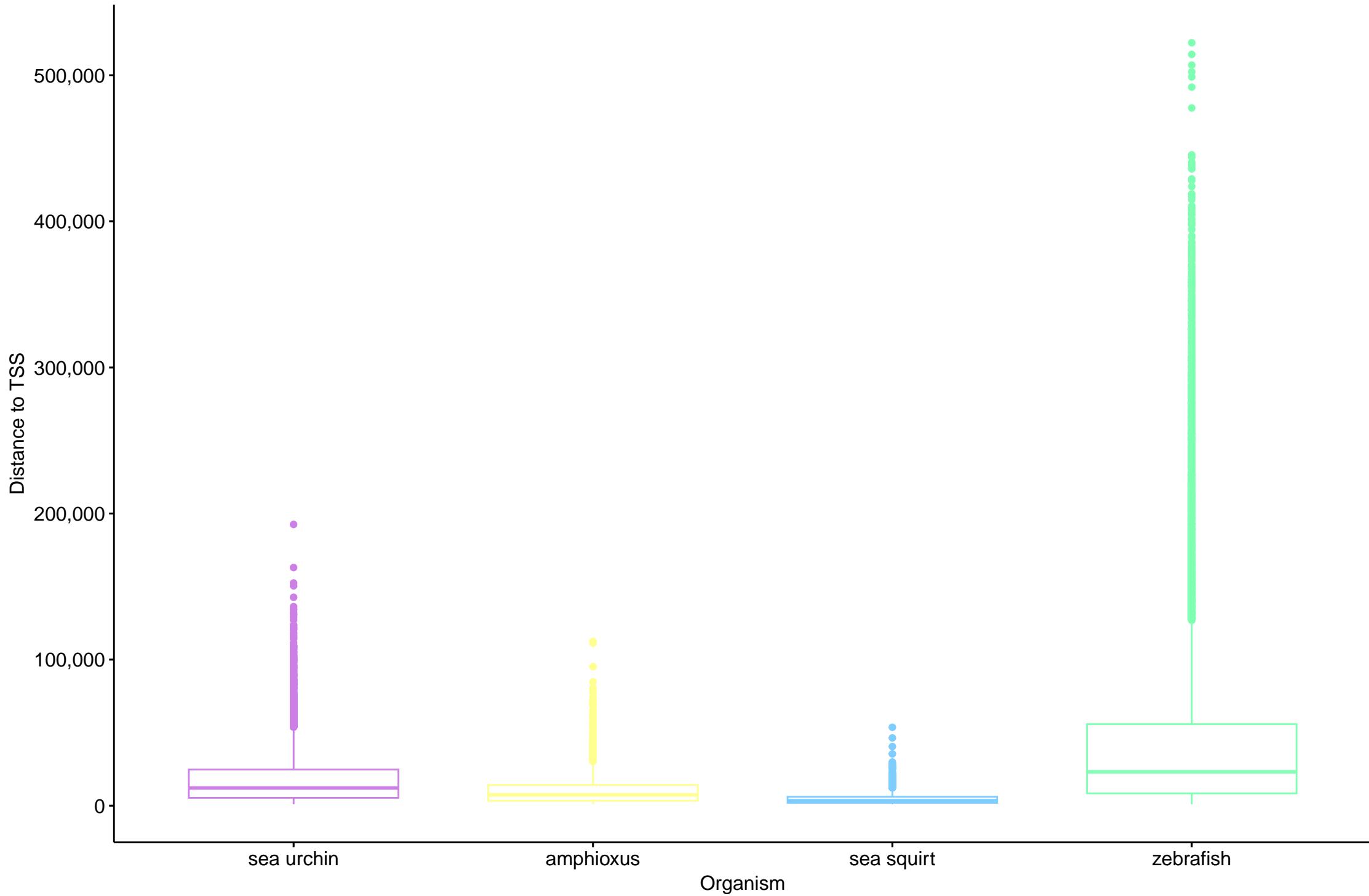

Supplement: Supplementary file 1 [file cells-13-01121-s001.zip › Supplementary_Figure_S1.pdf]
